# Circulating Tumor Cells Detected Using EpAb3-5 as Prognostic Indicators in Head and Neck Squamous Cell Carcinoma

**DOI:** 10.7150/jca.131794

**Published:** 2026-04-08

**Authors:** Po-Chih Hsu, Chun-Fan Lung, Chan-Yen Kuo, Han-Chung Wu, Yi-Chiung Hsu

**Affiliations:** 1 Department of Dentistry, Taipei Tzu Chi Hospital, Buddhist Tzu Chi Medical Foundation, New Taipei, Taiwan, R.O.C.; 2 Institute of Oral Medicine and Materials, College of Medicine, Tzu Chi University, Hualien 970, Taiwan, R.O.C.; 3 Department of Biomedical Sciences and Engineering, National Central University, Taoyuan, Taiwan, R.O.C.; 4 Institute of Cellular and Organismic Biology, Academia Sinica, Taipei 115, Taiwan, R.O.C.; 5 Center for Astronautical Physics and Engineering, National Central University, Taoyuan, Taiwan. R.O.C.; 6 Department of Medical Research, Cathay General Hospital, Taipei, Taiwan, R.O.C.

**Keywords:** head and neck squamous cell carcinoma (HNSCC), circulating tumor cells (CTC), EpAb3-5, prognostic biomarker

## Abstract

**Background:**

EpAb3-5, an anti-EpCAM (epithelial cell adhesion molecule) neutralizing antibody, enhances circulating tumor cell (CTC) detection, but its clinical and prognostic relevance in head and neck squamous cell carcinoma (HNSCC) remains unclear.

**Methods:**

We retrospectively analyzed 66 HNSCC patients. CTCs were enumerated using EpAb3-5 and a commercial EpCAM-based platform (MACS), and tumor EpCAM expression detected using EpAb3-5 was assessed by immunohistochemistry. Associations with clinicopathologic features were evaluated, and survival outcomes were analyzed using Kaplan-Meier and Cox regression models, truncated at five years.

**Results:**

EpAb3-5 detected significantly more CTCs than MACS (p < 0.001), indicating greater detection sensitivity. Tumor EpCAM expression detected by EpAb3-5 correlated with nodal status (p < 0.01) but not T classification or overall stage. Although EpAb3-5-detected EpCAM expression alone was not an independent predictor of overall survival (OS) or disease-free survival (DFS), its integration with tumor staging revealed a context-dependent prognostic association. Specifically, low EpCAM expression assessed using EpAb3-5 identified early-stage patients with better OS (p < 0.05), whereas no significant survival differences were observed in advanced-stage disease.

**Conclusions:**

EpAb3-5 enhances CTC detection compared with a conventional EpCAM-based method and provides complementary prognostic information when interpreted alongside tumor staging. These findings indicate that EpCAM immunoreactivity assessed using EpAb3-5 may assist in refining risk stratification in early-stage HNSCC; however, these observations should be considered exploratory and warrant further validation in larger prospective cohorts.

## Introduction

Head and neck squamous cell carcinoma (HNSCC) remains a significant global health burden. According to GLOBOCAN 2020 estimates, HNSCC accounted for approximately 890,000 new cases and 450,000 deaths annually, representing about 4.5% of all cancer diagnoses and deaths worldwide 1. Trend analyses using advanced modeling methods predict that the global incidence of HNSCC will rise by nearly 30%, surpassing 1 million new cases annually by 2030 2. Despite advances in multimodality treatment approaches such as surgery, radiotherapy, concurrent chemoradiation, and immunotherapy, 5-year survival rates remain modest, particularly for patients diagnosed with advanced-stage disease 3. This limited progress reflects the complexity of HNSCC, driven by its molecular heterogeneity and varied tumor biology. These variations can lead to markedly different prognoses, even among patients with similar clinicopathological characteristics, underscoring the need for more precise prognostic tools.

Recent studies in HNSCC have underscored the critical role of molecular biomarkers in refining patient prognosis, thereby complementing traditional clinical staging systems 4, 5. Several molecular and transcriptomic studies have further demonstrated that integrating biomarker information with clinical staging may improve prognostic stratification in HNSCC 6, 7. In addition, a range of molecular markers, including Eph family receptors 8, tumor-associated antigens such as squamous cell carcinoma antigen 9, epidermal growth factor receptor (EGFR) signaling components 10, and human papillomavirus (HPV) status 11, have been associated with survival outcomes in HNSCC, further highlighting the growing importance of molecular profiling in disease prognostication.

In recent years, circulating tumor cells (CTCs) have emerged as promising liquid biopsy biomarkers for disease monitoring in HNSCC 12. Multiple studies 13-15 and meta-analyses 16 have shown that CTC detection correlates with recurrence risk and survival outcomes in HNSCC. Molecular profiling studies further demonstrate that epithelial markers such as epithelial cell adhesion molecule (EpCAM) are commonly expressed on HNSCC CTCs, highlighting the importance of epithelial markers for CTC enrichment and detection 17. These findings emphasize the importance of appropriate epithelial markers for CTC enrichment and support the evaluation of EpAb3-5, an anti-EpCAM neutralizing antibody, as a tool for identifying EpCAM-positive CTCs that may correlate with tumor aggressiveness.

EpCAM expression, as detected using EpAb3-5 or related antibodies, has been associated with prognostic significance across multiple malignancies. In lung cancer, EpCAM overexpression plays a pivotal role in tumor cell survival and is linked to aggressive phenotypes and unfavorable outcomes 18. In colorectal cancer, EpCAM has also been implicated in tumor progression and metastatic potential 19, 20. Recent evidence in HNSCC also supports EpCAM's prognostic relevance, with high EpCAM expression correlating with significantly poorer overall survival (OS) in patients treated with definitive radiotherapy (2-year OS ~62% vs. ~88%, *p* < 0.05) and serving as an independent adverse prognostic factor in multivariate analysis 21. Moreover, emerging genomic data indicate that EpCAM mutations occur across epithelial cancers, including HNSCC 22. While their association with reduced survival and tumor progression has been established in cancers such as colon and hepatocellular carcinoma, corresponding evidence in HNSCC remains limited. Notably, EpCAM expression in HNSCC may correlate with poorer clinical outcomes, particularly in OS and disease-free survival (DFS), yet this relationship has not been clearly defined.

In light of the increasing emphasis on molecular biomarkers in HNSCC, this study first evaluates the ability of EpAb3-5 to improve CTC detection compared with a conventional EpCAM-based platform. We then assess the clinical relevance of EpCAM expression in tumor tissues as detected using EpAb3-5 and determine its association with OS and DFS. Furthermore, we investigate whether EpAb3-5-assessed EpCAM expression may provide complementary prognostic information when integrated with tumor staging. Through this analysis, our study seeks to clarify the potential role of EpCAM-targeted assessment in prognostic stratification of HNSCC.

## Materials and Methods

### Study design and patient cohort

This study was designed as a retrospective analysis of HNSCC patients to evaluate the prognostic value of EpCAM expression assessed using EpAb3-5 in combination with tumor stage on OS and DFS. A total of 66 HNSCC patients treated at Taipei Tzu Chi Hospital were included in the study. The inclusion criteria for the study population were as follows: (1) histologically confirmed diagnosis of HNSCC, (2) available clinical data on tumor staging according to the TNM (tumor, node, metastasis) system and survival outcomes, and (3) availability of tumor tissue samples for EpCAM expression analysis assessed using EpAb3-5. Additional clinical information, including primary tumor site and p16 immunohistochemistry (IHC) results, was retrieved from the hospital registry. The variables available in the study dataset are summarized in Table S1. The study protocol was reviewed and approved by the Institutional Review Board (IRB) of Taipei Tzu Chi Hospital (IRB No. 13-IRB027).

### CTC detection and quantification

CTCs were isolated from patient blood samples and labeled with cytokeratin 18 (green) as a marker of epithelial-origin cells. Nuclei were counterstained with DAPI (blue) for visualization. To compare antibody performance, CTCs were stained with the anti-EpCAM neutralizing antibody EpAb3-5 and with a commercial anti-EpCAM antibody (MACS, magnetic-activated cell sorting). Fluorescence microscopy was used to capture images of stained cells, and image-based enumeration was performed to quantify CTC counts detected by each antibody.

### Biomarker and tumor stage assessment

EpCAM expression in tumor tissues was assessed using EpAb3-5 by IHC. Archived formalin-fixed paraffin-embedded tumor samples were assembled into tissue microarrays. Sections of 4 μm thickness were stained with EpAb3-5, and two independent pathologists, blinded to clinical data, evaluated staining intensity using a semi-quantitative scoring system. A predefined cutoff score of 5 was applied to dichotomize patients into high and low EpCAM expression groups, as assessed using EpAb3-5. In this semi-quantitative scoring system, a score ≥5 corresponded to moderate-to-strong membranous staining in a substantial proportion of tumor cells and was used to define a higher EpCAM expression category for subsequent analyses. This threshold also yielded a reasonably balanced distribution of cases between groups, thereby reducing potential instability associated with extreme group imbalance. Tumor stage was determined using the TNM classification system (American Joint Committee on Cancer, AJCC). Patients were stratified into early-stage (I/II) and advanced-stage (III or higher) groups based on clinical stage at diagnosis.

### Statistical analysis

For CTC detection analysis, the Wilcoxon rank-sum test was used to compare CTC counts detected by EpAb3-5 and MACS in order to assess differences in detection efficiency. The Kruskal-Wallis test was conducted to evaluate variations in CTC counts across different overall cancer stages (I-IV). To examine the correlation between CTC counts and disease progression, patients were stratified into subgroups according to tumor staging (T1-T2 vs. T3-T4), nodal status (N0 vs. N1-N3), and clinical stage (Stage I-II vs. Stage III-IV), and the Wilcoxon rank-sum test was applied to assess significant differences in CTC counts between these groups. Kaplan-Meier survival curves were generated to assess the impact of EpCAM expression assessed using EpAb3-5 and tumor stage on OS and DFS. OS was defined as the time from diagnosis to death from any cause, and DFS was defined as the time from diagnosis to the first occurrence of disease recurrence. Survival curves were compared using the log-rank (Mantel-Cox) test between high and low EpCAM expression (EpAb3-5 IHC) groups (cutoff value = 5) and between early-stage (Stage I-II) and advanced-stage (Stage III-IV) groups. For conditional subgroup analyses, patients were categorized using combined criteria that incorporated both tumor staging parameters and EpCAM expression (EpAb3-5 IHC) levels. Specifically, subgroups were defined by multiple “AND” conditions, such as low EpCAM expression with early T stage (T1-T2), or high EpCAM expression with advanced nodal status (N1-3). Kaplan-Meier survival curves were then generated for each combined subgroup, and survival differences were evaluated using the log-rank (Mantel-Cox) test. For the quantification of survival risks associated with EpCAM expression (high vs. low, as assessed using EpAb3-5), overall tumor stage, T stage, and N stage, Cox proportional hazards regression models were employed. Hazard ratios (HRs) and 95% confidence intervals (CIs) were calculated to estimate the relative risk of OS and DFS within each grouping category. All statistical analyses were conducted using R (version 4.4.1) and the “survival” and “survminer” package. Statistical significance was set at *p* < 0.05 across all analyses. All survival analyses were truncated at five years (1,825 days), with events occurring beyond this time censored to ensure comparability across patients with differing follow-up durations.

## Results

### EpAb3-5 demonstrates higher sensitivity for CTC detection compared with MACS

**Figure 1:**
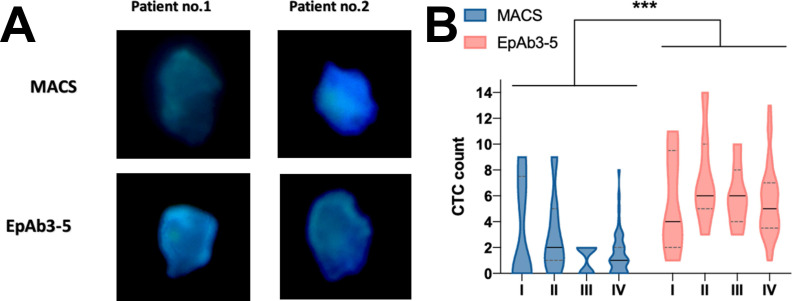
** Circulating tumor cell (CTC) detection in HNSCC patients.** (A) CTCs labeled with cytokeratin 18 (green) and counterstained with DAPI for nuclei (blue).(B) Quantitative comparison of CTC counts detected by EpAb3-5 and the commercial anti-EpCAM (epithelial cell adhesion molecule) antibody (MACS, magnetic-activated cell sorting) across tumor stages I-IV. Statistical comparisons were performed using the Mann-Whitney U test. *** indicates *p* < 0.001.

To evaluate the detection efficiency of EpAb3-5 in CTC analysis, we first compared its performance with MACS, a widely used commercial anti-EpCAM antibody. Representative fluorescence microscopy images from two patients (Figure 1A) show that both antibodies identified epithelial-origin CTCs counterstained with DAPI, whereas EpAb3-5 produced consistently stronger fluorescence signals. Quantitative analysis further demonstrated that EpAb3-5 detected significantly more CTCs than MACS across tumor stages I-IV (Figure 1B, *p* < 0.001).

Collectively, these results indicate that EpAb3-5 demonstrated higher detection sensitivity for CTC detection relative to MACS, supporting its potential utility as an improved EpCAM detection tool. Based on this technical validation, we next examined the clinicopathologic and prognostic associations of EpCAM expression assessed using EpAb3-5 in tumor tissues.

### Distribution of primary tumor site and p16 status in the study cohort

Additional clinical information regarding primary tumor site and p16 IHC status was retrieved from the hospital registry. All patients included in the present cohort had primary tumors located in the oral cavity (100%). Among the available records, only one patient (1.5%) showed p16 positivity, while the remaining patients were p16-negative.

### EpCAM expression assessed by EpAb3-5 is associated with nodal status in HNSCC

**Figure 2:**
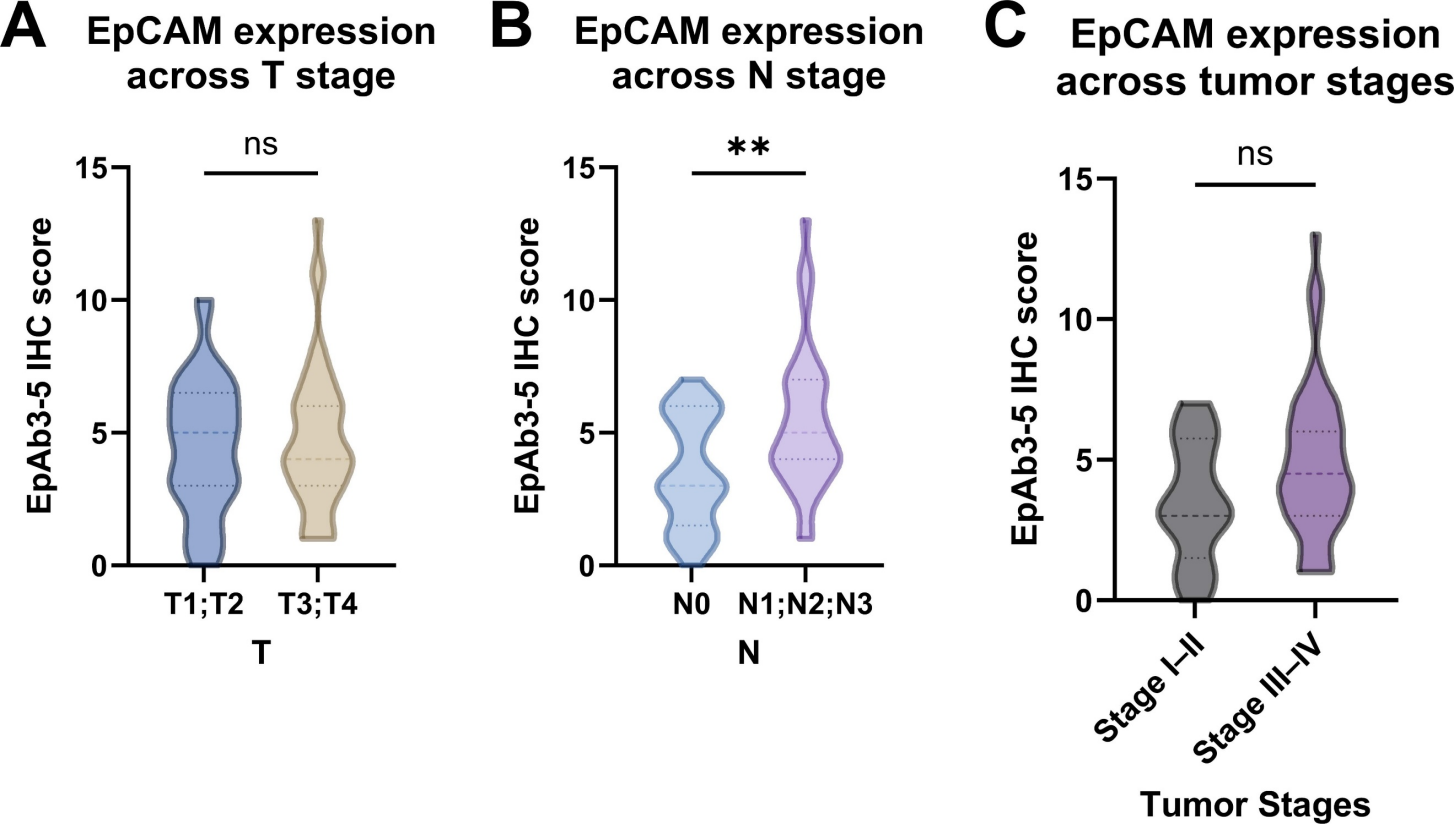
Comparison of epithelial cell adhesion molecule (EpCAM) expression determined by EpAb3-5 immunohistochemistry (IHC) across tumor characteristics in HNSCC patients. (A) Primary tumor size (T1-T2 vs. T3-T4). (B) Nodal status (N0 vs. N1-3). (C) Overall tumor stage (Stage I-II vs. Stage III-IV). Statistical comparisons were performed using the Wilcoxon rank-sum test. ** indicates *p* < 0.01; ns, not significant.

Building on the improved sensitivity of EpAb3-5 in CTC detection compared with MACS, we next investigated whether EpCAM expression detected using EpAb3-5 in tumor tissues was associated with established clinicopathologic factors. When patients were stratified by primary tumor size, no significant difference in EpCAM expression (EpAb3-5 IHC) was observed between early T stage (T1-T2) and advanced T stage (T3-T4) (Figure 2A). In contrast, a significant association emerged with nodal involvement: patients with positive nodal status (N1-3) exhibited higher EpCAM expression (EpAb3-5 IHC) compared with those without nodal metastasis (N0) (Figure 2B, *p* < 0.01). Analysis according to overall clinical stage showed a trend toward higher EpAb3-5-assessed EpCAM expression in advanced-stage disease (Stage III-IV) compared with early-stage disease (Stage I-II), but this difference did not reach statistical significance (Figure 2C). Taken together, these findings suggest that EpAb3-5-assessed EpCAM expression did not show significant differences according to primary tumor size or overall clinical stage, but may be selectively enriched in patients with nodal metastasis, which may indicate an association with regional disease progression.

### EpAb3-5-assessed EpCAM expression shows a trend toward prognostic association in the overall HNSCC cohort

Having established that EpAb3-5 offers higher sensitivity for CTC detection compared with MACS and that its tumor tissue expression is associated with nodal status, we next evaluated the prognostic relevance of EpCAM expression (EpAb3-5 IHC) and tumor stage on patient survival. Kaplan-Meier analysis demonstrated that OS was significantly worse in patients with advanced T stage (T3-T4) compared with those with early T stage (T1-T2) (Figure S1A, *p* < 0.05). Similarly, nodal involvement (N1-3) was associated with poorer OS relative to node-negative disease (N0) (Figure S1B, *p* < 0.05). A trend toward reduced OS was also observed in advanced-stage disease (Stage III-IV) compared with early-stage disease (Stage I-II), though this difference did not reach statistical significance (Figure S1C, *p* = 0.055). When patients were stratified by EpCAM expression (EpAb3-5 IHC), those with high expression (≥ 5) showed a tendency toward better OS compared with the low-expression group (< 5), although the difference was not statistically significant (Figure S1D, *p* = 0.15). Analysis of DFS yielded comparable findings. Patients with advanced T stage exhibited a trend toward poorer DFS compared with those with early T stage (Figure S1E, *p* = 0.056), while nodal status and overall clinical stage did not show significant associations with DFS (Figure S1F, *p* = 0.29; Figure S1G, *p* = 0.14). High EpAb3-5-assessed EpCAM expression was associated with a trend toward improved DFS, but again this did not achieve statistical significance (Figure S1H, *p* = 0.085).Together, these findings indicate that traditional staging parameters, particularly T and N categories, remain strong predictors of OS in HNSCC, whereas EpAb3-5-assessed EpCAM expression alone shows only a non-significant trend toward improved survival outcomes in the overall cohort. These findings indicate that the prognostic association of EpCAM expression assessed using EpAb3-5 may vary according to disease context.

### Prognostic sensitivity of EpAb3-5-assessed EpCAM expression emerges in early-stage HNSCC

**Figure 3:**
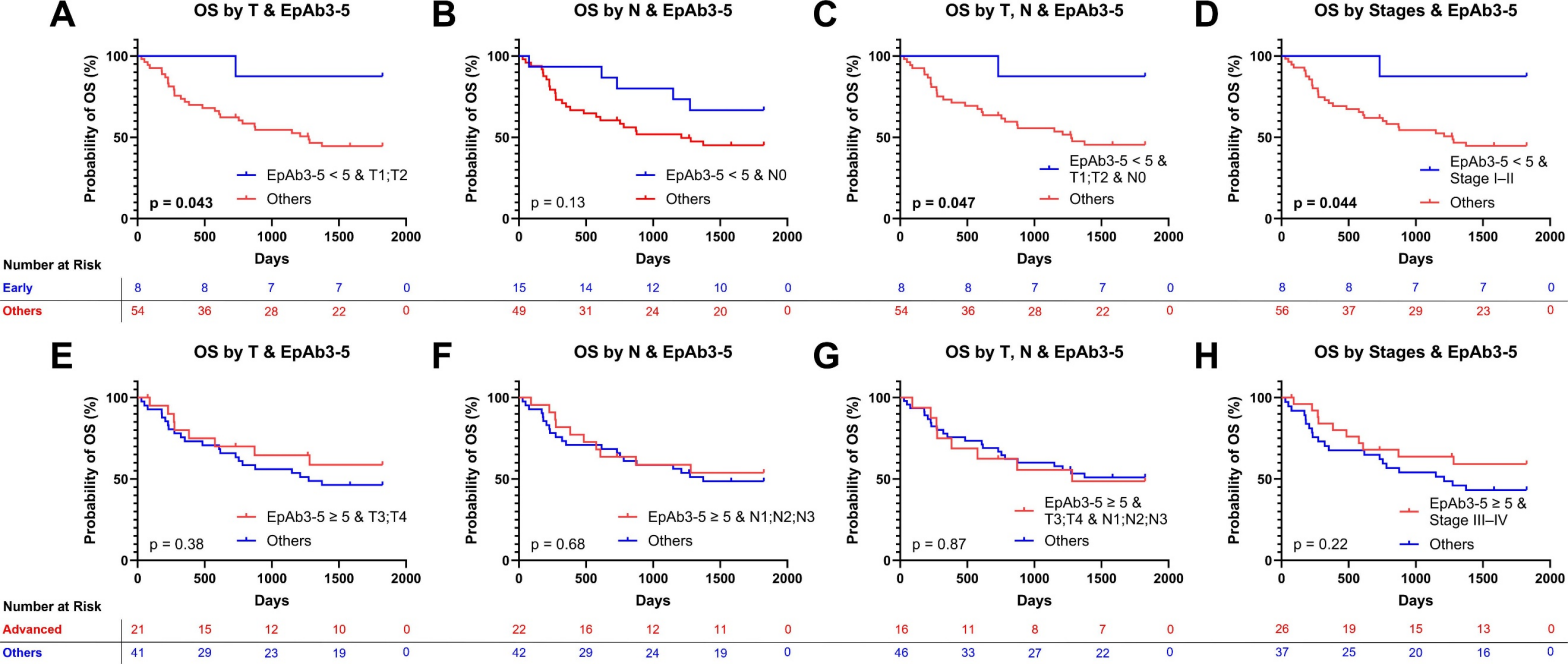
** Overall survival (OS) analysis using combined EpAb3-5-assessed epithelial cell adhesion molecule (EpCAM) expression and tumor staging conditions in HNSCC patients.** (A-D) OS curves comparing patients with low EpCAM expression (< 5, assessed using EpAb3-5) in early-stage categories [T1-T2 (A), N0 (B), combined T1-T2 and N0 (C), and Stage I-II (D)] against all other patients. (E-H) OS curves comparing patients with high EpCAM expression (≥ 5, assessed using EpAb3-5) in advanced-stage categories [T3-T4 (E), N1-3 (F), combined T3-T4 and N1-3 (G), and Stage III-IV (H)] against all other patients. Kaplan-Meier survival curves were generated and compared using the log-rank test. All analyses were truncated at five years (1,825 days), with events beyond this time censored. Statistical significance was determined using the log-rank (Mantel-Cox) test, with significant p-values shown in bold.

Although EpCAM expression (EpAb3-5 IHC) alone showed only a trend toward prognostic association in the overall cohort, we next examined whether its impact became clearer when integrated with established clinicopathologic factors. Using multiple “AND” conditions, patients were grouped by both EpCAM expression (EpAb3-5 IHC) levels and tumor staging parameters (T, N, or overall stage).Among patients with early-stage features, low EpAb3-5-assessed EpCAM expression (< 5) was associated with significantly better OS. This benefit was evident in those with early T stage (T1-T2; Figure 3A, *p* < 0.05), combined T1-T2 and N0 disease (Figure 3C, *p* < 0.05), and Stage I-II classification (Figure 3D, *p* < 0.05), with a similar but non-significant trend in node-negative patients (Figure 3B, *p* = 0.13). In contrast, these same subgroups showed only nonsignificant trends toward improved DFS, including T1-T2 (Figure S2A, *p* = 0.28), N0 (Figure S2B, *p* = 0.77), combined T1-T2 and N0 (Figure S2C, *p* = 0.28), and Stage I-II (Figure S2D, *p* = 0.29).By comparison, patients with advanced disease features and high EpCAM expression (≥ 5, assessed using EpAb3-5) did not show survival advantages in either OS or DFS. OS outcomes were similar across advanced T stage (T3-T4; Figure 3E, *p* = 0.38), nodal involvement (N1-3; Figure 3F, *p* = 0.68), combined T3-T4 with N1-3 (Figure 3G, *p* = 0.87), and Stage III-IV disease (Figure 3H, *p* = 0.22). Likewise, no significant differences were observed for DFS across these corresponding advanced subgroups (Figures S2E-H). To evaluate the robustness of the predefined cutoff, sensitivity analyses were performed using alternative EpAb3-5 thresholds across a plausible range (4-7). Across these thresholds, the direction of the association between low EpCAM expression assessed using EpAb3-5 and favorable OS in early-stage subgroups remained consistent, with comparable survival patterns observed for cutoffs of 4, 6, and 7 (Figure S3). Collectively, these findings indicate that the prognostic association of EpCAM expression assessed using EpAb3-5 is context-dependent. Low EpAb3-5-assessed EpCAM expression was associated with improved long-term survival in a subset of early-stage patients, particularly for OS, whereas high expression did not provide additional prognostic discrimination in advanced-stage disease. Importantly, this effect appears more pronounced for OS than for DFS, indicating that EpAb3-5 may be more relevant to predicting long-term survival outcomes than recurrence risk in HNSCC.

### Cox regression underscores nodal status and suggests a predictive role for EpAb3-5 in early-stage HNSCC

Building on the subgroup analyses showing context-dependent prognostic associations of EpAb3-5-assessed EpCAM expression, we next evaluated the independent contributions of tumor staging parameters and EpCAM expression using multivariable Cox regression models for both OS and DFS.

For OS (Table 1), nodal involvement (N1-3 vs. N0) showed the strongest effect, with an estimated HR of 2.70 (95% CI, 0.92-7.93; *p* = 0.071), indicating a higher risk of death in patients with nodal metastasis. EpCAM expression according to EpAb3-5 IHC scoring demonstrated an HR of 0.51 (95% CI, 0.23-1.13; *p* = 0.097). Although the HR was below 1.0, the association did not reach statistical significance. Neither T stage (HR, 1.51; *p* = 0.599) nor overall clinical stage (HR, 1.12; *p* = 0.917) showed significant associations with OS.

For DFS (Table 2), similar patterns were observed. High EpCAM expression (≥ 5, assessed using EpAb3-5) was associated with an HR of 0.59 (95% CI, 0.29-1.19; *p* = 0.141). By contrast, T stage (HR, 1.93; *p* = 0.391), N stage (HR, 1.43; *p* = 0.397), and overall stage (HR, 0.87; *p* = 0.882) did not significantly predict DFS.

As a whole, these Cox regression analyses support the established prognostic importance of nodal status in HNSCC. To evaluate whether this single p16-positive case influenced the observed associations, a sensitivity analysis was performed after excluding that patient. The Cox regression results for both OS and DFS remained essentially unchanged (Tables S2 and S3). While EpCAM immunoreactivity assessed using EpAb3-5 did not emerge as an independent predictor of either OS or DFS, subgroup analyses indicated differential trends in early-stage disease when combined with conventional staging parameters.

## Discussion

This study investigated the clinical significance of EpAb3-5-assessed EpCAM expression and its detection sensitivity for CTCs in patients with HNSCC. Our results show that EpAb3-5 detected more CTCs than MACS, a commercial anti-EpCAM antibody (Figure 1). In the present study, this comparison primarily served as a methodological validation step to highlight the higher EpCAM detection capability of EpAb3-5 relative to a conventional platform. Based on this technical validation, we subsequently explored the clinicopathologic and prognostic associations of EpCAM expression assessed using EpAb3-5 in tumor tissues.

While EpCAM expression detected using EpAb3-5 did not emerge as an independent prognostic marker for OS or DFS in the overall cohort (Figure S1), its combination with tumor staging parameters revealed a context-dependent prognostic pattern (Figure 3). Specifically, patients with low EpAb3-5-assessed EpCAM expression and early-stage features showed better five-year OS, suggesting that EpCAM expression may provide additional stratification value when interpreted alongside established clinicopathologic factors. Importantly, although Cox regression analyses showed hazard ratios below 1.0 for higher EpCAM expression, these associations did not reach statistical significance (Tables 1 and 2). Rather than indicating a protective effect, these findings reinforce the observation that the prognostic relevance of EpCAM expression appears to be context-dependent, becoming more apparent when combined with tumor staging parameters in early-stage disease (Figure 3).Notably, the prognostic association was more evident for OS than for DFS. In early-stage patients, low EpAb3-5-assessed EpCAM expression was significantly associated with improved OS in most early-stage subgroup analyses (Figure 3A, 3C, and 3D), whereas only a nonsignificant trend was observed in node-negative patients (Figure 3B). In contrast, the corresponding DFS analyses showed only nonsignificant trends across these subgroups (Figure S2A-D). This difference may reflect the distinct clinical implications of these endpoints. DFS primarily captures the timing of initial recurrence, whereas OS integrates not only recurrence but also subsequent disease progression, treatment response, and survival after relapse. The stronger association with OS may therefore suggest that EpCAM expression assessed using EpAb3-5 is more closely related to tumor aggressiveness, systemic progression, or post-recurrence survival, including potential differences in response to salvage therapy, rather than solely to the risk of first recurrence. In addition, the limited number of DFS events within early-stage subgroups may have reduced statistical power to detect significant differences. Further studies with larger cohorts and detailed analyses of recurrence patterns are needed to clarify whether EpCAM expression preferentially influences late-stage progression or post-recurrence survival dynamics. These findings are supported by prior studies that have linked EpCAM expression to tumor aggressiveness and poor prognosis in HNSCC. For instance, research has shown that elevated EpCAM expression is associated with inferior outcomes following radiotherapy 21, underscoring its potential role in resistance mechanisms. Furthermore, studies of CTCs in HNSCC have reported that their presence is associated with recurrence and poorer survival outcomes. For example, a prospective study demonstrated that baseline CTC positivity predicted both recurrence and cancer-related death, with a significantly increased risk of recurrence (odds ratio 8.40; p < 0.0001) 13. A systematic review and meta-analysis further confirmed that CTC positivity is linked to shorter DFS (pooled HR 4.62; 95% CI 2.51-8.52) 16. Associations between CTC burden and nodal status, however, appear more heterogeneous: in one large cohort, CTC counts correlated with nodal stage only in p16-positive OPSCC but not consistently across all HNSCC patients 13. In this context, our finding that EpAb3-5-detected CTCs are more prevalent in patients with nodal involvement (N1-3) supports the potential of EpAb3-5 as a relevant detection marker in the metastatic setting.

Beyond detection, the molecular role of EpCAM in HNSCC progression adds biological interpretability to our findings. EpCAM signaling intersects with key oncogenic pathways such as EGFR, and its cleaved extracellular domain has been shown to interfere with EGFR-driven epithelial-mesenchymal transition and resistance to targeted therapies like cetuximab 23. Although this study does not directly assess mechanistic pathways, the elevated EpCAM staining signal detected using EpAb3-5 in advanced-stage patients may reflect underlying tumor biology characterized by enhanced epithelial features or increased tumor shedding.

Our findings also echo emerging research into therapeutic targeting of EpCAM. Neutralizing antibodies such as EpAb2-6 have shown preclinical activity in reducing PD-L1 expression and inducing tumor apoptosis 24, supporting the clinical relevance of this biomarker family. While EpAb3-5 is not yet used therapeutically, its dual role in tissue IHC and blood-based CTC detection positions it as a promising candidate for integrated diagnostic or monitoring applications.

Nevertheless, several limitations warrant consideration. The study is retrospective and single-center in design, with a modest sample size that limits statistical power, particularly for subgroup analyses and multivariable modeling. The five-year survival truncation, while ensuring consistency in follow-up, may underestimate long-term survival patterns. Additionally, EpCAM expression was evaluated using a predefined cutoff based on EpAb3-5 IHC scoring; although alternative scoring approaches yielded consistent survival trends (Figure S3), data-driven optimization or external validation in larger cohorts may further refine its clinical applicability. Importantly, although p16 IHC data were retrieved from the hospital registry, only one patient in the cohort was p16-positive. Because of this extremely low prevalence, p16 status could not be meaningfully incorporated into the multivariable Cox regression models. However, sensitivity analyses excluding this case produced nearly identical results (Tables S2 and S3), suggesting that the overall findings were unlikely to be substantially confounded by p16 status. Nevertheless, because HPV-related HNSCC is known to have distinct biological behavior and more favorable survival outcomes 25, the predominance of p16-negative tumors in this cohort should be considered when interpreting the survival associations observed in this study. In addition, treatment heterogeneity may still introduce residual confounding. Finally, since the CTC enumeration technique relied on fluorescence microscopy rather than standardized platforms, its reproducibility across studies may be limited.

In sum, the use of EpAb3-5 enhances the sensitivity of CTC detection compared with a conventional commercial antibody. Although EpCAM expression assessed using EpAb3-5 was not an independent prognostic factor in the overall cohort, it demonstrated context-dependent significance when integrated with tumor staging. In early-stage disease, low EpAb3-5-assessed EpCAM expression was associated with more favorable outcomes, indicating its potential value in refining prognostic stratification beyond traditional staging systems. From a clinical perspective, such stratification may help identify subsets of early-stage patients with more favorable prognosis, potentially informing individualized follow-up strategies and treatment planning. These findings support the interpretation of EpAb3-5 not as a stand-alone marker but as a complementary biomarker that may improve risk assessment in well-defined patient subsets. Future work should focus on prospective multi-center studies with larger sample sizes, standardized detection protocols, and inclusion of molecular factors such as HPV status to validate its clinical relevance. In addition, longitudinal monitoring of EpAb3-5-positive CTCs and mechanistic studies of its role in tumor progression and treatment resistance could advance its translation from a research tool into a clinically applicable biomarker, which may ultimately contribute to more precise and personalized management of HNSCC patients.

## Conclusion

EpAb3-5 enhances the sensitivity of CTC detection compared with a conventional commercial antibody and provides additional context-dependent prognostic information when evaluated alongside established clinicopathologic factors. Although EpAb3-5-assessed EpCAM expression alone does not serve as an independent predictor of OS or DFS, its integration with tumor staging identifies early-stage subgroups with more favorable outcomes, supporting the role of EpCAM expression as a complementary biomarker for refining risk stratification and potentially informing clinical management, including follow-up strategies and treatment decision-making in HNSCC. These findings suggest that EpAb3-5 may contribute to improved prognostic assessment when used in conjunction with traditional staging systems, pending further validation in larger prospective studies.

## Supplementary Material

Supplementary figures and tables.

## Figures and Tables

**Table 1:** Cox regression analysis of grouping indicators and their association with five-year overall survival (n = 62).

Grouping Indicator	HR	CI (Lower)	CI (Upper)	p-value
T1;T2 vs. T3;T4	1.505974	0.327527	6.924491	0.598884
N0 vs. N1;N2;N3	2.701004	0.920077	7.929142	0.07055
Tumor Stage I;II vs. III;IV	1.121132	0.129896	9.676476	0.91719
EpAb3-5-assessed EpCAM expression < vs. ≥ 5	0.514431	0.234755	1.1273	0.096793

HR = Hazard Ratio, CI = Confidence Interval, EpCAM = Epithelial Cell Adhesion Molecule.

**Table 2:** Cox regression analysis of grouping indicators and their association with five-year disease-free survival (n = 60).

Grouping Indicator	HR	CI (Lower)	CI (Upper)	p-value
T1;T2 vs. T3;T4	1.934507	0.428514	8.733253	0.390879
N0 vs. N1;N2;N3	1.429222	0.625704	3.264603	0.39677
Tumor Stage I;II vs. III;IV	0.869912	0.138757	5.453766	0.881712
EpAb3-5-assessed EpCAM expression < vs. ≥ 5	0.585402	0.287036	1.193911	0.140871

HR = Hazard Ratio, CI = Confidence Interval, EpCAM = Epithelial Cell Adhesion Molecule.

## Abbreviations

HNSCC: head and neck squamous cell carcinoma
OS: overall survival
DFS: disease-free survival
EGFR: epidermal growth factor receptor
HPV: human papillomavirus
EpCAM: epithelial cell adhesion molecule
IHC: immunohistochemistry
HR: hazard ratio
CI: confidence interval
CTC: circulating tumor cell
MACS: magnetic-activated cell sorting
