# Supplementary material for: Circulating Tumor Cells Detected Using EpAb3-5 as Prognostic Indicators in Head and Neck Squamous Cell Carcinoma
Source: J Cancer. 2026 Apr 8;17(4):881–9. doi: 10.7150/jca.131794 (PMC13105145; doi:10.7150/jca.131794)
Supplement: Supplementary file 1 — Supplementary figures and tables. [file jcav17p0881s1.pdf]

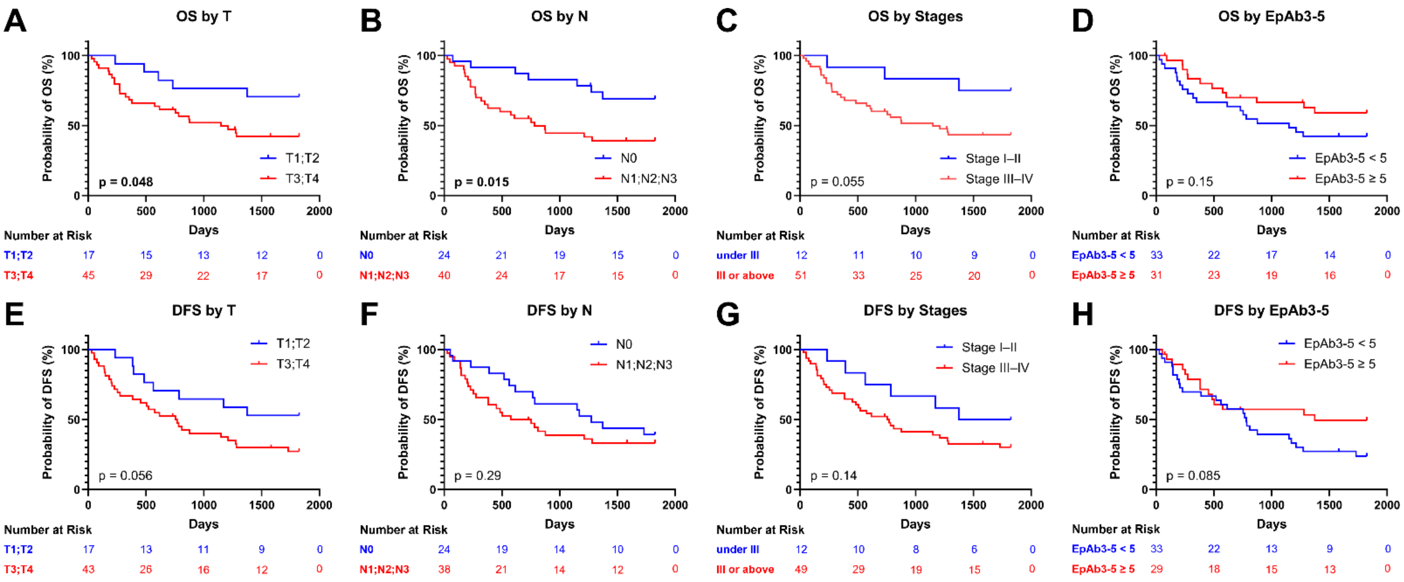

Figure S1 Kaplan-Meier analysis of overall survival (OS) and disease-free survival (DFS) in HNSCC patients.

(A–C) OS stratified by T stage (T1–T2 vs. T3–T4), nodal status (N0 vs. N1–3), and overall clinical stage (Stage I–II vs. Stage III–IV).

(D) OS stratified by epithelial cell adhesion molecule (EpCAM) expression (cutoff < 5 vs. ≥ 5, assessed using EpAb3-5).

(E–G) DFS stratified by T stage, nodal status, and overall clinical stage.

(H) DFS stratified by EpCAM expression (cutoff < 5 vs. ≥ 5, assessed using EpAb3-5).

Survival curves were generated using the Kaplan-Meier method and compared with the log-rank test. All analyses were truncated at five years (1,825 days), with events beyond this point censored. Statistical significance was determined using the log-rank (Mantel-Cox) test, with significant p-values shown in bold.

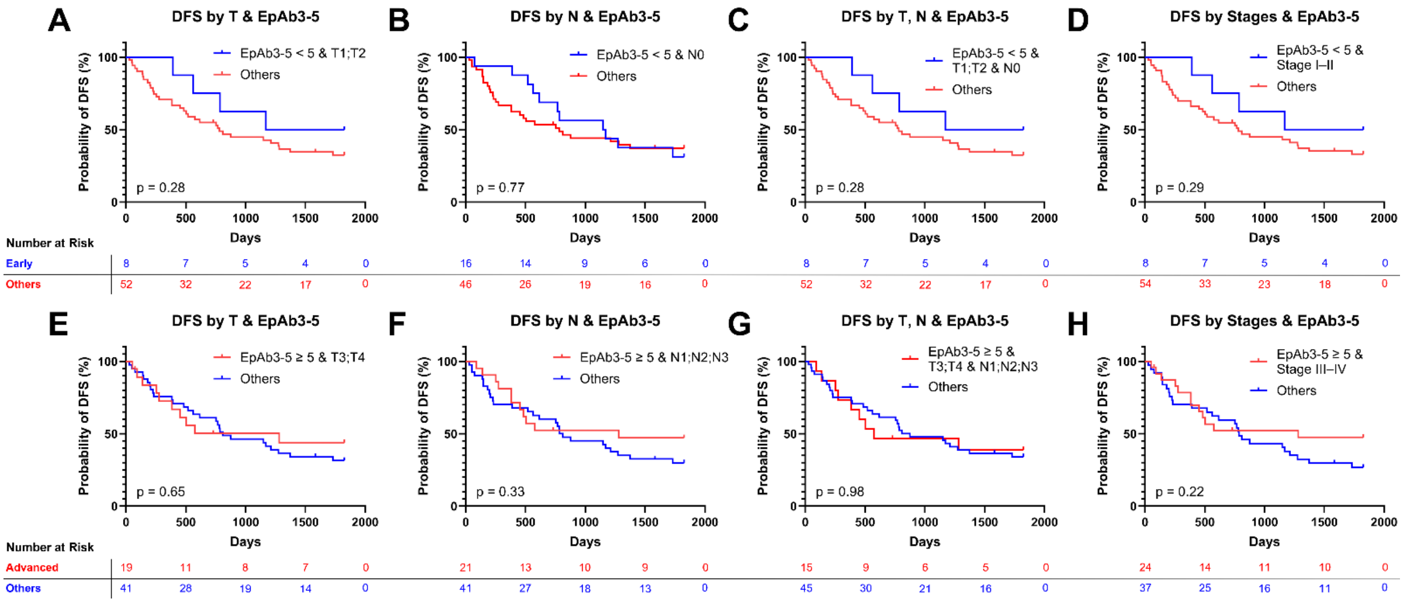

Figure S2 Disease-free survival (DFS) analysis using combined EpAb3-5–assessed epithelial cell adhesion molecule (EpCAM) expression and tumor staging conditions in HNSCC patients.

(A–D) DFS curves comparing patients with low EpCAM expression (< 5, assessed using EpAb3-5) in early-stage categories [T1–T2 (A), N0 (B), combined T1–T2 and N0 (C), and Stage I–II (D)] against all other patients.

(E–H) DFS curves comparing patients with high EpCAM expression (≥ 5, assessed using EpAb3-5) in advanced-stage categories [T3–T4 (E), N1–3 (F), combined T3–T4 and N1–3 (G), and Stage III–IV (H)] against all other patients.

Kaplan-Meier survival curves were generated and compared using the log-rank test. All analyses were truncated at five years (1,825 days), with events beyond this time censored. Statistical significance was determined using the log-rank (Mantel-Cox) test, with significant p-values shown in bold.

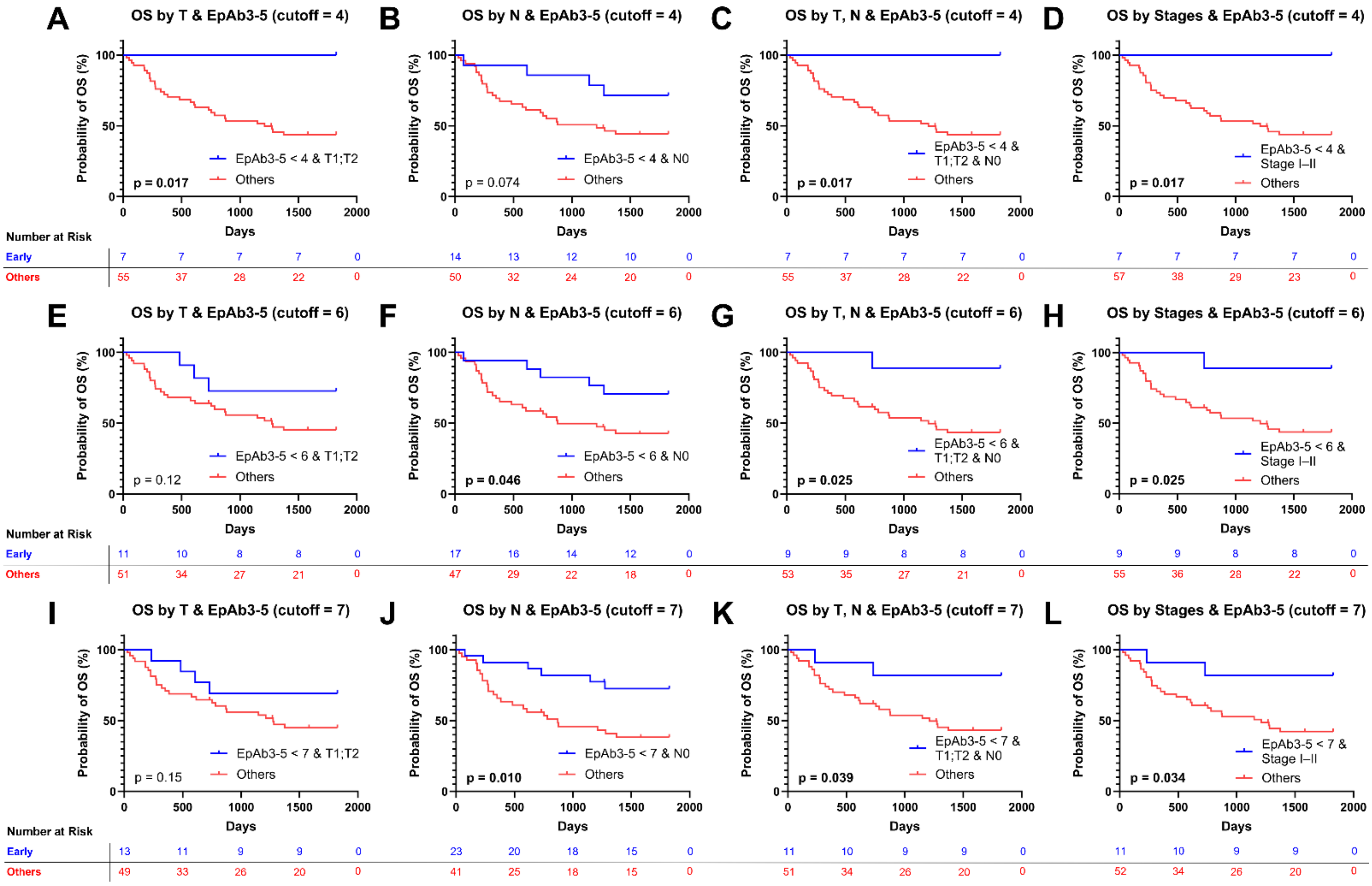

Figure S3 Sensitivity analysis of overall survival (OS) using alternative EpAb3-5 cutoffs in early-stage HNSCC.

(A–D) cutoff = 4; (E–H) cutoff = 6; (I–L) cutoff = 7.

Within each cutoff set, panels correspond respectively to T1–T2, N0, T1–T2 & N0, and Stage I–II. Survival curves were compared using the log-rank (Mantel–Cox) test and truncated at five years (1,825 days), with events beyond this time censored.

Table S1 Summary of variables available in the study dataset.

Variable	Description	Type / Coding
**Patient number**	Unique identifier assigned to each patient	Integer
**Primary tumor site**	Anatomical location of the primary tumor	Categorical
**TNM classification**	Tumor stage according to the TNM system	T, N, M categories
**Clinical stage**	Overall stage grouping based on TNM classification	Stage I–IV
**p16 status**	p16 IHC result used as an HPV surrogate marker	Positive / Negative
**CTC count (EpAb3-5)**	CTC count detected using the EpAb3-5 anti-EpCAM antibody	Integer
**CTC count (MACS)**	CTC count detected using the MACS EpCAM-based platform	Integer
**OS days**	Time from diagnosis to death or last follow-up	Continuous
**OS status**	OS event indicator	1 = death, 0 = censored
**DFS days**	Time from diagnosis to recurrence, death, or last follow-up	Continuous
**DFS status**	DFS event indicator	1 = recurrence or death, 0 = censored

IHC = **I**mmuno**h**isto**c**hemistry, HPV = **H**uman **P**apilloma**v**irus, CTC = **C**irculating **T**umor **C**ell, EpCAM = Epithelial Cell Adhesion Molecule, MACS = **M**agnetic-**A**ctivated **C**ell **S**orting, OS = **O**verall **S**urvival; DFS = **D**isease-**f**ree **S**urvival.

Table S2 Cox regression analysis of grouping indicators and their association with five-year overall survival after exclusion of the p16-positive patient (n = 61).

Grouping Indicator	HR	CI (Lower)	CI (Upper)	p-value
**T1;T2 vs. T3;T4**	1.509053	0.328140	6.939857	0.597104
**N0 vs. N1;N2;N3**	2.672531	0.905079	7.891489	0.075169
**Tumor Stage I;II vs. III;IV**	1.118321	0.129577	9.651700	0.919000
**EpAb3-5–assessed EpCAM expression < vs. ≥ 5**	0.517090	0.233560	1.144807	0.103851

HR = Hazard Ratio, CI = Confidence Interval, EpCAM = Epithelial Cell Adhesion Molecule.

Table S3 Cox regression analysis of grouping indicators and their association with five-year disease-free survival after exclusion of the p16-positive patient (n = 59).

Grouping Indicator	HR	CI (Lower)	CI (Upper)	p-value
**T1;T2 vs. T3;T4**	1.926671	0.426713	8.699207	0.393848
**N0 vs. N1;N2;N3**	1.424164	0.618175	3.281016	0.406321
**Tumor Stage I;II vs. III;IV**	0.871640	0.139007	5.465577	0.883395
**EpAb3-5–assessed EpCAM expression < vs. ≥ 5**	0.583795	0.283911	1.200434	0.143390

HR = Hazard Ratio, CI = Confidence Interval, EpCAM = Epithelial Cell Adhesion Molecule.
